# Brazilian immigrants’ oral health literacy and participation in oral health care in Canada

**DOI:** 10.1186/s12903-016-0176-1

**Published:** 2016-02-15

**Authors:** Paola Calvasina, Herenia P. Lawrence, Laurie Hoffman-Goetz, Cameron D. Norman

**Affiliations:** Oswaldo Cruz Foundation- FIOCRUZ, Young Talent Scientist Fellowship CAPES, Ceará, Brazil; Department of Biological and Diagnostic Sciences, Discipline of Dental Public Health, Faculty of Dentistry, University of Toronto, Toronto, ON Canada; School of Public Health and Health Systems, University of Waterloo, Waterloo, ON Canada; Dalla Lana School of Public Health, University of Toronto, Toronto, ON Canada

**Keywords:** Oral health literacy, Immigrants, Information-seeking behaviour, Decision making, Dental care

## Abstract

**Background:**

Inadequate functional health literacy is a common problem in immigrant populations. The aim of this study was to investigate the association between oral (dental) health literacy (OHL) and participation in oral health care among Brazilian immigrants in Toronto, Ontario, Canada.

**Methods:**

The study used a cross-sectional design and a convenience sample of 101 Brazilian immigrants selected through the snowball sampling technique. Data were analyzed using descriptive statistics and logistic regression modeling.

**Results:**

Most of the sample had adequate OHL (83.1 %). Inadequate/marginal OHL was associated with not visiting a dentist in the preceding year (OR = 3.61; *p* = 0.04), not having a dentist as the primary source of dental information (OR = 5.55; *p* < 0.01), and not participating in shared dental treatment decision making (OR = 1.06; *p* = 0.05; OHL as a continuous variable) in multivariate logistic regressions controlling for covariates. A low average annual family income was associated with two indicators of poor participation in oral health care (*i.e*., not having visited a dentist in the previous year, and not having a dentist as regular source of dental information).

**Conclusion:**

Limited OHL was linked to lower participation in the oral health care system and with barriers to using dental services among a sample of Brazilian immigrants. More effective knowledge transfer will be required to help specific groups of immigrants to better navigate the Canadian dental care system.

## Background

Health literacy (HL) is an important social determinant of health and a key to understanding patients’ health-related behaviours [1]. Limited HL has been linked to: i) difficulty accessing and making sense of health information [2]; ii) lower interest in participating in health care decision making [3, 4]; iii) less compliance with preoperative medication instructions [5], and; iv) greater use of emergency health care services [6].

The links established between HL and health-related behaviours have led to greater interest in developing oral health literacy (OHL) concepts and instruments to narrow the gap in understanding the needs and abilities of individuals or communities with respect to oral health care needs [7–12]. OHL has been described as the “degree to which individuals have the capacity to obtain, process and understand basic oral health information and services needed to make appropriate health decisions” ([13] p.21). It includes cognitive and social skills required to access, understand, evaluate and improve oral health in a variety of settings across the life course.

Immigrants are at higher risk of poor health [14] and may have lower OHL [11] compared to non-immigrant populations. Although researchers have associated HL to health-related behaviours among many North-American immigrant groups [15–18], very few studies have been published on the OHL of immigrants [19–21], and none has explored the relationships between OHL skills and participation in oral health care.

This exploratory study applies Ishikawa & Yano’s [1] conceptual framework to investigate the association between OHL skills and indicators of participation in oral health care among a sample of Brazilian immigrants in Toronto, Ontario (ON), Canada. Health communication studies have identified behaviours such as health care service use, information seeking, and participation in decision making as indicators of active participation in medical care [1], [22], [23]. Thus, in the present study, we use these same indicators to assess participation in oral health care, including: 1) dental health care service utilization (time since last dental visit), 2) dental information seeking (dentist as the usual source of dental information), and 3) dental treatment decision making (active participation in their own dental treatment decisions). We hypothesized that limited functional OHL is associated with a lower participation of Brazilian immigrants in the oral health care process.

## Methods

A convenience sample of 101 Brazilian immigrants living in Toronto, ON was recruited from April to June 2010. In the absence of published data that specifically addressed our study objectives, we estimated the sample size based on Sabbahi et al.’s [10] study. The sample size needed to give a precision of 1/4 standard deviation for the Oral Health Literacy Instrument (OHLI) score (i.e., SD 13.9 × 0.25 = 3.475) based on a 95 % confidence interval was approximately 62; for a precision of 1/5 standard deviation, 97 participants were needed. Therefore, we planned to recruit about 100 participants using snowball sampling.

Initially, the sampling criteria consisted of: 1) born in Brazil, 2) 18 years or older, 3) Portuguese was the first or primary language, 4) have lived in Toronto, Canada for >1 and <5 years, and 5) willing to be interviewed and complete a survey. Eligible participants with documented cognitive or mental impairment were excluded. Random sampling was not adopted because no reliable sampling frame existed for Brazilian immigrants in Toronto. According to the 2006 Canada Census data, Brazilian immigrants represent less than 1 % (0.12 %, approximately 7000 people) of the Greater Toronto Area (GTA) population and are geographically dispersed. Therefore, participants were recruited using a two-phase strategy: (1) advertising to recruit an initial sample; (2) respondent-driven (snowball) sampling technique to increase the sample size, if 100 participants were not recruited in the first phase [24]. Cooperation in recruitment was sought from local agencies providing social services for immigrants. The following were used to augment recruitment: Craigslist, Facebook, word-of-mouth and flyers in local Brazilian communities in Toronto. This recruitment phase led to approximately 20 participants contacting the primary investigator for interview. Additional individuals were recruited by snowballing [24]. Despite the initial inclusion criteria, 21 participants with >5 years living in the GTA were also recruited because the majority of recent immigrants residing in Canada for less than 5 years had never been to a dentist in Canada and would not be able to answer questions related to any dental care experience in the country.

Eligible participants completed a one-hour long self-administered questionnaire. All instruments and questionnaire items were translated from English to Portuguese and then back-translated to English following standard techniques [25]. Attention was paid to cross-cultural adaptation of the instruments. One translator independently translated the instruments from English to Portuguese; then another translator, totally blind to the original versions, did the reverse translations. Once these translations were completed, the original instruments in English and the back-translated versions were compared by a panel of two translators and one expert familiar with these kinds of survey. Participants could choose to answer the questionnaire in English or Portuguese.

Ethical review and research approval was obtained from the University of Toronto Research Ethics Board. All participants provided informed consent prior to participating in the survey, and each received an incentive of $30.

### Measures

Survey data were collected in eight domains: participants’ socio-demographic characteristics, oral health-related behaviors, self-rated oral health, OHL, oral health knowledge, oral self-efficacy, health literacy, and oral health-related quality of life.

Socio-demographic characteristics of the sampled immigrants included: age, sex, education (highest level of education and whether the participant pursued studies in Canada), employment status, time living in Canada and average family income. The questionnaire also included items on self-rated oral health and dental care needs, access to dental insurance and barriers to dental treatment (Table 1).

**Table 1:** Characteristics of the study sample

Mean age (SD)	34.5 (9)
Female % (n)	73.3 (74)
College/University Education vs. High School or less % (n)	82.2 (83)
Pursued studies in Canada % (n)	77.2 (78)
Had a job last month % (n)	83.2 (84)
Average family income (CAD/year)	
< $15,000–$30,000	47.5 (48)
$31,000–$70,000	38.6 (39)
> $71,000	12.9 (13)
Less than 5 years living in Canada vs. 5+ years % (n)	79.2 (80)
Excellent/very good self-rated oral health vs. good/fair/poor % (n)	77.2 (78)
Self-reported untreated dental conditions % (n)	52.5 (53)
Access to, and participation in, the oral health care process	
Primary source of oral health information % (n)	
Dentist	67.3 (68)
Internet	23.8 (24)
TV/Radio	8.9 (9)
Dental treatment decision making % (n)	
Wants to be involved in the decision-making process vs. does not/don’t know	93.1 (94)
Participates fully in decisions about his/her dental treatment vs. does not/leaves treatment decisions to the dentist	20.8 (21)
Health care service use % (n)	
Visited a dentist in Canada or Brazil since time of arrival	60.4 (61)
Visited a dentist <1 year ago vs. 1+ years ago	75.2 (76)
Usually treats a toothache in a private dental office vs. public dental clinic or hospital	85.1 (86)
Presence of barrier(s) to dental treatment vs. no barrier(s)	42.6 (43)
Cost is the main dental care barrier vs. other barrier(s)	28.7 (29)
Pays directly out-of-pocket for dental care vs. employment health insurance, provincial/municipal program coverage	56.4 (57)
Median/Mean OHLI^a^ (SD)	85.5/83.4 (10)
Median/Mean Oral Health Knowledge^a^ (SD)	64.7/62.2 (19.4)
Median/Mean OHES^b^ (SD)	4.0/3.9 (0.63)
Median/Mean OHIP-14^c^ (SD)	14.0/14.1 (9.6)
Oral health literacy^a^ % (n)	
Adequate	83.1 (84)
Marginal	13.9 (14)
Inadequate	3 (3)
Oral health knowledge^a^ % (n)	
Adequate	29.7 (30)
Marginal	23.8 (24)
Inadequate	46.5 (47)
Health literacy^d^ % (n)	
Adequate	56.4 (57)
Inadequate	43.6 (44)
Preferred language of test %(n)	
Portuguese	86.1 (87)
English	13.9 (14)

^a^Oral Health Literacy Instrument (OHLI) and Oral Health Knowledge scale by Sabbahi *et al*. [10]

^b^Oral Health Empowerment Scale (OHES) adapted from Anderson *et al*. [24]

^c^Brazilian version of the Oral Health Impact Profile – Short Form (OHIP-14) [26]

^d^Chew *et al*. [25]

OHL was measured using the Oral Health Literacy Instrument (OHLI) [10]. This reading comprehension and numeracy instrument has demonstrated good concurrent and construct validity, high internal consistency (Cronbach’s alpha >0.7), and good test-retest reproducibility (ICC >0.6). The OHLI is scored on a scale of 0–100: ≥75 rates adequate, 60–74 marginal, and ≤59 inadequate OHL [10]. The internal consistency for the Portuguese version of OHLI was tested during the study, and results were also high (Cronbach’s alpha = 0.84).

Oral health knowledge was assessed using a validated 17 labelled-items test [10]. Scores range from 0 to 100: 0–59, 60–74, and 75–100 were categorized as inadequate, marginal and adequate oral health knowledge, respectively [10].

To assess oral health self-efficacy, we adapted a self-efficacy measure validated for research on diabetes [26], and developed an Oral Health Empowerment Scale (OHES). Where “diabetes” was mentioned in the original tool, it was replaced with “oral health”. OHES uses an eight-item questionnaire and assesses the individual’s confidence in taking an active role in the management of his/her condition (e.g., obtaining social support, managing stress, etc.). It uses five-point Likert-type responses from “strongly disagree” to “strongly agree”. The higher the score the higher the patient empowerment. Completed item responses were summed and averaged to obtain an overall continuous OHES score. This instrument showed high internal consistency (Cronbach’s alpha = 0.77).

Immigrants’ health literacy was obtained from a validated single, five-point Likert type question: “How often do you have problems learning about your medical condition because of difficulty understanding written information?” (responses ranged from “always” to “never”) [27]. Oral health-related quality of life was examined using the previously validated Brazilian version of the short-form of the Oral Health Impact Profile scale (OHIP-14) with higher scores indicating greater impact of oral conditions on quality of life [28].

Outcome variables were three indicators of participation in oral health care: 1) dental health care service utilization, 2) dental information seeking, and 3) dental treatment decision making. Dental health care service utilization was assessed using the time of the respondents’ last dental visit with a three-category response (<1 year ago; 1–2 years ago; more than 2 years ago), dichotomized at a 1-year cut-off level for analysis. Dental information seeking was measured via the following question: “Where do you learn the most information about oral health?”. Responses allowed for multiple answers, and were coded “1” for “from my dentist”, and “0” for all other responses, including “I do not seek information about oral health”, “from other health professionals”, “from TV/Radio/newspaper/magazines”, “internet”, and “others, specify”.

Dental treatment decision making was initially assessed by a four-item response: “I do not participate in my dental treatment decisions and do not intend to”, “I do not participate in my dental treatment decisions but I intend to”, “I participate to some extent”, and “I fully participate in my dental treatment decisions”. The first two items were subsequently classified as “does not participate in the process of dental decision making”, and the last two items were categorized as “does participate in dental decision making”.

### Data analysis

Frequency distributions were tabulated for socio-economic data, demographics and access to, and participation in, oral health care. In addition, both mean and medians were tabulated for all the scales used in this study since graphical and statistical evaluation of OHES and Oral Health Knowledge scales showed a relatively normal distribution, while OHLI was negatively skewed and OHIP-14 was positively skewed. After descriptive statistics were conducted, we assessed whether measures of OHL were related to participation in the oral health care process variables and to the covariates. OHL was dichotomized into adequate vs. inadequate/marginal for bivariate analyses of factors associated with inadequate/marginal OHL using the Pearson Chi-squared test or Fisher’s Exact test [interpreted at the 5 % significance level]. The strength of the associations was described with Odds Ratios (ORs) and their 95 % confidence intervals (CIs). Lastly, logistic regression models were used to test the association between OHL with each of the three indicators of participation in oral health care, while controlling for covariates in the analyses. The modeling strategy used stepwise selection with the significance level boundary for variable entry and variable removal set to 10 %. Data were analyzed using SPSS version 19.0. Two-tailed statistical tests were used.

## Results

Participants’ characteristics are shown in Table 1. Participants were aged between 18 and 57 (mean 34.5, SD ± 9). Most were highly educated (82.2 %) and had adequate OHL (83.1 %); scores for the OHLI ranged from 45 to 99 (mean 83.4, SD 10). Nearly half (47.5 %) reported an average family income of less than 30,000.00 CAD per year and the majority of participants have been living in Canada for less than 5 years (79.2 %).

The factors statistically significantly associated with inadequate/marginal OHL in bivariate analyses included: the participants’ level of education; whether they were currently studying in Canada; if they used dental services for emergency care only or for regular care; citing language or cultural barriers to getting dental treatment done in Canada; and relying on the dentist as the primary source of oral health information (data not tabulated). Briefly summarized, having a college or university degree was associated with a 79 % decrease in odds for inadequate/marginal OHL compared with those with a High School education or less [OR = 0.21, 95 % Confidence Interval (CI) 0.07–0.68]. Being in Canada for reasons other than pursuing further education increased the odds for inadequate/marginal OHL by 409 % (OR = 4.09, 95 % CI = 1.36–12.3). In addition, episodic users of dental services were nearly five times more likely to have inadequate/marginal OHL than those who visited the dentist once a year or more often in the previous year (OR = 4.96, 95 % CI = 1.43–17.24). Although 77.2 % of participants rated their oral health as “excellent/very good” (Table 1), seven participants reported experiencing severe toothache in the past month. Among barriers to accessing services, which included lack of dental insurance, inability to afford dental treatment and language or cultural barriers, those with language barriers (*n* = 7) were six times as likely as those without such barriers to have limited OHL (OR = 6.10, 95 % CI = 1.11–33.56). Dental information seeking behaviors such as “always” checking the expiration date and leaflets of medication or asking the dentist about oral health care were associated with OHL. Specifically, those who did not use a dentist as their primary source of oral health information were 5.2 times more likely to have inadequate/marginal OHL (OR = 5.17, 95 % CI = 1.71–15.64).

### OHL and dental service utilization

A logistic regression model identified two significant correlates of dental services use less than 1 year prior to the study, when covariates were controlled for in the analysis (Table 2). Participants with an average family income between $15,000 and $30,000 per year, compared with those with > $71,000/year (OR = 3.67; *p* = 0.07), and those with marginal/inadequate OHL vs. adequate (OR = 3.61; *p* = 0.04), were more likely to have visited a dentist >1 year ago. The resulting Nagelkerke pseudo-*R*^2^ value of the model containing these two variables was 0.167.

**Table 2:** Final logistic regression model evaluating participation in oral health care by time since last dental visit (*n* = 101)

Outcome: dental visit more vs. less than one year ago	Model 1 β(SE)	Adjusted OR^a^ (95 % CI)	*P*-value
Average annual family income			
>$71,000	REF	REF	
$31,000–$70,000	0.23 (0.76)	1.26 (0.28–5.64)	0.76
$15,000–$30,000	1.30 (0.73)	3.67 (0.87–15.46)	0.07
Oral health literacy			
Adequate	REF	REF	
Marginal/inadequate	1.28 (0.60)	3.61 (1.10–11.87)	0.04

β (logistic regression coefficient); (SE) (standard error); Odds ratio (OR); (95 % CI) 95 % Confidence Interval

^a^Odds ratios adjusted for age, education, time living in Canada, self-rated oral health, oral health-related quality of life, oral health knowledge, medical health literacy, and oral health self-efficacy

### OHL and dental information seeking

Two main factors were associated with not having the dentist as the usual source of dental information; namely, average family income and OHL (Table 3). Controlling for covariates, including access to dental care, participants with an average family income per year between $15,000 and $30,000, compared to respondents with income > $71,000/year, were 10.55 times more likely to not have a dentist as the usual source of dental information (*p* = 0.03). Participants with marginal/inadequate OHL compared to participants with adequate OHL were 5.55 times more likely to not have a dentist as the usual source of dental information (*p* < 0.01). Together these variables yielded a Nagelkerke pseudo *R*^2^ of 0.251 in the dental information-seeking model.

**Table 3:** Final logistic regression model evaluating participation in oral health care by dental information seeking (*n* = 101)

Outcome: usual source of dental information (not from a dentist vs. from a dentist)	Model 2 β(SE)	Adjusted OR^a^ (95 % CI)	*P*-value
Average annual family income			
>$71,000	REF	REF	
$31,000–$70,000	1.26 (1.14)	3.54 (0.38–32.84)	0.27
$15,000–$30,000	2.36 (1.11)	10.55 (1.20–92.49)	0.03
Oral Health Literacy			
Adequate	REF	REF	
Marginal/Inadequate	1.72 (0.63)	5.55 (1.62–19.05)	<0.01

β (logistic regression coefficient); (SE) (standard error); Odds ratio (OR); (95 % CI) 95 % Confidence Interval

^a^Odds ratios adjusted for age, time living in Canada, self-rated oral health, oral health knowledge, oral health-related quality of life, medical health literacy, oral health self-efficacy, access to dental care

### OHL and dental treatment decision making

In the final logistic regression model evaluating dental treatment decision-making behavior, OHLI scores (continuous variable) were associated with respondents’ active participation in dental treatment decision making (Table 4). That is, for every unit increase in the OHLI scale, the adjusted odds ratio for participation in dental treatment decision making increased by 6 %. The Nagelkerke pseudo *R*^2^ was 0.097.

**Table 4:** Final logistic regression model evaluating participation in oral health care by dental-treatment decision-making behaviour (*n* = 101)

Outcome: participation in dental treatment decision making (yes vs. no)	Model 3 β(SE)	Adjusted OR^a^ (95 % CI)	*P*-value
OHLI (continuous)	0.60 (0.02)	1.06 (1.01–1.11)	0.05

β (logistic regression coefficient); (SE) (standard error); Odds ratio (OR); (95 % CI) 95 % Confidence Interval

^a^Odds ratio adjusted for age, education, time living in Canada, self-rated oral health, oral health knowledge, oral health-related quality of life, medical health literacy, oral health self-efficacy, oral health care use, and answering Questionnaire in English

## Discussion

This investigation examined the relationship between OHL and outcomes of participation in oral health care among a convenience sample of Brazilian immigrants. We found support for the hypothesized association between OHL and participation in the health care system. Brazilian immigrants with limited OHL were more likely to have visited a dentist more than 1 year ago, to obtain dental information from sources other than dentists, and to participate poorly in any process of shared dental treatment decision making. The important role of OHL for participation in oral health care supports the application of this conceptual model to develop health education interventions in community and clinical settings. Increased OHL may help individuals understand their problem, seek information from various sources, and make informed shared decisions that would lead to better treatment adherence and subsequent oral health self-management.

The Brazilian immigrants enrolled in our study had a high level of OHL on average. At least two possible explanations exist for these high OHL rates. First, our sampling strategy might have led participants with a high level of education to enrol in our study. More than 80 % of respondents reported completing at least a college education in Brazil, all of whom scored significantly higher on the OHL test than those with less than High School completion. This finding is consistent with studies indicating that level of education is a strong predictor of both HL [15] and OHL [10, 12, 14]. Second, participants were given the option to complete the questionnaire in English or Portuguese; 86.1 % selected the Portuguese version, which potentially suggests lack of confidence in English comprehension. Previous studies have clearly shown that on average, immigrants are more confident in understanding medical information [29, 30] and perform better on medical comprehension tests in their first language [15, 16].

This study identified barriers for Brazilian immigrants to participate in the process of dental care. For example, among participants who had already visited a dentist in Canada, 93.1 % wanted to be informed of any treatment options and be part of the decision-making process. However, only 20.8 % reported active full participation in dental treatment decisions. This discrepancy might be explained by limited English proficiency or limited OHL, including the ability to comprehend oral health information and a lack of self-confidence among immigrant groups in addressing their oral health needs [18], [29].

Not surprisingly, average annual family income was correlated with barriers to dental utilization and dental information seeking. Participants with an average family income of < $30,000 a year were almost four times more likely to not have visited a dentist within the previous year. Access to dental care and dental utilization for the overall adult population in Canada is highly dependent on socioeconomic status, insurance coverage, and employment benefits [31]. Our findings suggest increased disparities in access to oral health care among highly educated Brazilian immigrants with potential negative consequences on their oral health. Prior to immigrating, it is possible that study participants did not experience such barriers to utilizing dental services regularly. Upon arriving in Canada, however, the decline in social status and income resources of immigrants might significantly limit dental utilization. It should also be noted that some of the participants in this study reported travelling back to Brazil in order to tend to their dental care needs because dental care costs were actually more affordable back home. Although these findings were qualitative, transnational dental care utilization has recently been reported among Canadian immigrants. It is estimated that approximately 11,500 immigrants have used dental care outside Canada between 2001 and 2005 [32]. Taken together, these findings may explain the importance of income as a determinant of utilization of dental services among new immigrant populations.

These results should be considered in light of the study’s limitations. First, the adopted sampling strategy, resulting in a convenience sample, restricts generalizability. Furthermore, the low number of participants with marginal or inadequate OHL resulted in some large confidence intervals and may have affected the power to detect other significant differences. As a consequence, conclusions may only be extended to groups with characteristics similar to the sample studied (Table 1 should be used in any comparative analysis).

Second, the cross-sectional study design prevents establishing a causal pathway between immigrants’ limited OHL skills and their poor participation in the oral health care process. Future research using longitudinal designs is needed to ascertain whether limited OHL causes poor dental utilization or poor utilization causes limited OHL. Nevertheless, the associations presented here provide a basis for future research on OHL conceptualization and measurement with other English-as-a-second-language immigrant groups.

Third, although the Portuguese version of the OHLI was cross-culturally validated and pilot tested and showed high internal consistency, reproducing this instrument in other studies would require further psychometric validation. Similarly, the OHES instrument was developed purposefully for this study.

Fourth, this study originally planned to include immigrants who had lived in Toronto for >1 and <5 years, but the high proportion of recent Brazilian immigrants who had never been to a dentist in Canada surprised us. We therefore enrolled immigrants who lived in Canada for >5 years, feeling that length of residency would not substantially affect the relationship between immigrants’ OHL and participation in oral health care. Indeed, time since immigration was not correlated with any outcome measure in this study.

## Conclusion

This study underscores the policy implications that both financial and non-financial factors exert on poor participation in oral health care among this immigrant group. We suggest that this specific group of Brazilian immigrants to Canada face challenges to access and navigate the Canadian dental care system that are brought about by low income, language barriers and lack of self-efficacy/knowledge about the dental system. The poor participation in oral health care in lower income groups might be dealt with through policies supporting better economic integration of immigrants. Also, it may be necessary for governments to strengthen specialized language and citizenship skill programs, in order for immigrants to learn the skills necessary to navigate the complexities of the Canadian health care system.

## Abbreviations

GTA: Greater Toronto Area
HL: health literacy
OHES: oral health empowerment scale
OHL: oral health literacy
OHLI: oral health literacy instrument
ON: Ontario
SPSS: statistical package for the social sciences
